# Molecular diet analysis of Anguilliformes leptocephalus larvae collected in the western North Pacific

**DOI:** 10.1371/journal.pone.0225610

**Published:** 2019-11-27

**Authors:** Seinen Chow, Nobuharu Inaba, Satoshi Nagai, Hiroaki Kurogi, Yoji Nakamura, Takashi Yanagimoto, Hideki Tanaka, Daisuke Hasegawa, Taiga Asakura, Jun Kikuchi, Tsutomu Tomoda, Taketoshi Kodama

**Affiliations:** 1 National Research Institute of Fisheries Science, Japan Fisheries Research and Education Agency, Kanazawa, Yokohama, Japan; 2 Civil Engineering Research Institute for Cold Region, Public Works Research Institute, Sapporo, Hokkaido, Japan; 3 Aquaculture Research Institute, Kindai University, Higashimuro, Wakayama, Japan; 4 Tohoku National Fisheries Research Institute, Japan Fisheries Research and Education Agency, Shiogama, Miyagi, Japan; 5 RIKEN Center for Sustainable Resource Science, Tsurumi, Yokohama, Japan; 6 Shibushi Station, National Research Institute of Aquaculture, Japan Fisheries Research and Education Agency, Shibushi, Kagoshima, Japan; University of Illinois at Chicago, UNITED STATES

## Abstract

Natural diets of leptocephalus larvae have been enigmatic. In this study, we collected DNA samples from the gut contents and body surface of leptocephali belonging to the five Anguilliform families (Anguillidae, Chlopsidae, Congridae, Muraenidae, and Serrivomeridae) from the northwest Pacific and performed next-generation 18S rDNA sequencing. Wide variety of eukaryotes was detected in both samples, from which eight eukaryotic groups (jellyfish, conoid parasite, tunicate, copepod, krill, segmented worm, fungi, and dinoflagellate) were selected on the basis of abundance. All groups except conoid parasites were common in both the samples. Cnidarian 18S rDNA reads were the most abundant in both the samples; however, the number of samples having cnidarian reads and the read counts were significantly higher in the body surface scraping samples than in the gut content samples, regardless of careful rinsing of the body surface. These results indicate that the cnidarian DNAs are most likely found because of cross contamination from the body surface and/or environment. 18S rDNA read counts of copepod and tunicate in the gut contents were greater than or comparable with those in the body surface scraping samples, which may correspond to the previous observations of fecal pellets and larvacean houses in the leptocephali gut. Thus, the present study supports previous implications that leptocephali utilize detritus materials, so called marine snow.

## Introduction

Marine and freshwater eels belonging to the order Anguilliformes have peculiar transparent leaf-like larvae called leptocephalus. Natural diets of the leptocephali have been enigmatic. Earlier studies have reported a poorly differentiated gut in the leptocephali containing amorphous and no identifiable food material [1–3]. Dissolved organic compounds were suspected to be directly absorbed through the epidermis, and it was hypothesized that the leptocephali utilized its sharp teeth to puncture other organisms and swallow the body fluid [2]. However, later histological investigation indicated that the alimentary tract of the Japanese eel (*Anguilla japonica*) leptocephali is functional for the uptake and digestion of intact macromolecules [4]. In captive condition, the wild-caught leptocephali of pike conger (*Muraenesox cinereus*) and white-spotted conger (*Conger myriaster*) showed no active preference toward zooplanktons, such as copepod larvae and ctenophore; however, these leptocephali visually located and actively engulfed squid paste [5]. Fecal pellets of zooplankton, aloricate protozoa, phytoplankton-like small spherical cells, and detrital aggregates have been observed in the gut of leptocephali [6–11], and the trophic positions of *C*. *myriaster* leptocephali have been observed to be nearly equal to the particulate organic matter [6].

Molecular analyses have been recently used to determine the gut contents of the leptocephali. Although 18S rDNA sequences from a wide variety of plankton organisms have been detected from the gut of the European eel (*A*. *anguilla*) leptocephali, gelatinous zooplankton (hydrozoan jellyfish) was suspected to be the major diet [12]. However, no animal ribosomal DNA (internal transcribed spacer 1) was found in the gut samples of the Japanese eel leptocephali, and already-degraded material was suspected in the diet [13]. Recently, a more advanced metagenomic analysis using next-generation sequencing (NGS) was applied, revealing that 76% of 18S rDNA reads recovered from the gut of European eel leptocephalus belongs to the phylum Cnidaria [14]. However, the consumption of cnidarian jellyfish contradicts with the results of stable isotope analysis, in which the trophic positions of the leptocephali have been reported to be low [6,15–19]. An inherent problem in the previous molecular studies [12–14] is that no specimen from the body surface of the leptocephali was analyzed, which could be a major source of cross contamination.

We performed 18S rDNA-based metagenomic analysis using NGS not only for the gut content samples but also for the body surface scraping samples of the leptocephali, in which cnidarian 18S rDNAs in the body surface scraping samples predominated over those in the gut content samples.

## Materials and methods

### Ethics statement

Larval samples captured with plankton nets deployed from research vessels were dead on retrieval and sampled at this time, and all plankton net operations were carried out in high seas outside the Exclusive Economic Zone. Therefore, the approval of coastal states was not required under the United Nations Convention on the Law of the Sea (UNCLOS).

### Leptocephali sampling and identification

Isaacs-Kidd Midwater Trawl (IKMT) net (8.7 m^2^ opening, 13 m long, 0.5 mm mesh, and canvas made cod-end) was used to collect the leptocephali. Oblique tows from the depth of 200 m to the surface were performed at night in the northwest tropical and subtropical Pacific from September to October of 2017. The leptocephali were sorted after collection and placed on ice-cold petri dish. Leptocephali having gut content (GC) were visually identified, and one side of the body surface was alternately rinsed three to four times using sterilized and refrigerated artificial sea water. Sterile swab was used to scrape the body surface (body surface scraping sample: BSS) and the tip of a swab was placed in separate sterile 1.5 mL Eppendorf tube. Subsequently, GC were squeezed out using sterilized inoculating loop and pipetted into separate sterile 1.5 mL Eppendorf tube. A small piece of the dorsal muscle was dissected and placed in a separate sterile 1.5 mL Eppendorf tube. All these tubes were kept at ‒60°C and transferred to the laboratory. DNA samples of the muscle, GC and BSS were extracted using a DNA extraction kit (Genomic Prep Cell and Tissue DNA Isolation Kit, Amersham Bioscience). DNA extracted from the muscle was used for partial amplification of mitochondrial 16S rDNA and nuclear 18S rDNA segments, in which a universal primer pair (16Sar-L and 16Sbr-H) [20] was used for the former and a primer pair (18S30F: 5’-GTCTCAAAGATTAAGCCATGC-3’ and 18S580R: 5’-CACCAGACTTGCCCTCCAAT-3’) for the latter. PCR amplification conditions are described previously [21] using annealing temperature of 55°C for the former and 58°C for the latter. Direct nucleotide sequencing for the amplified fragments was performed using the PCR primers.

### Peptide nucleotide acid (PNA) directed PCR clamping

We adopted PNA directed PCR clamping to selectively inhibit amplification of the host 18S rDNA [13,22]. A PNA probe was designed to anneal to the sequence near 5’ region in the 18S rRNA gene, and the nucleotide sequence was NH_2_-ACGGCCGGTACAGTG-CONH_2_ having 80.7°C Tm. Versatility of a primer pair for 18S rDNA mentioned above was tested by using a wide range of eukaryotes: Japanese eel (*A*. *japonica*), Japanese pufferfish (*Takifugu rubripes*), Japanese pilchard (*Sardinops melanostictus*), broadbanded thornyhead (*Sebastolobus macrochir*), Pacific bluefin tuna (*Thunnus orientalis*), freshwater shrimp (*Palaemon paucidens*), pronghorn spiny lobster (*Panulirus penicillatus*), long-spined sea urchin (*Diadema setosum*), brown macroalgae (*Sargassum horneri* and *Petalonia binghamiae*), diatom (*Phaeodactylum tricornutum*), and dinoflagellate (*Ceratoperidinium falcatum*), in which amplification of an expected size of fragment (c.a. 550 bp) was observed in all species. Efficiency of PCR clamping was tested by adding 1 μL PNA (10 μM) to 25 μL of PCR reaction mixture using eukaryote samples mentioned above. Efficient clamping was observed in Japanese eel, broadbanded thornyhead, and Pacific bluefin tuna, while no apparent inhibition of amplification was observed in the other organisms.

### Genetic analysis of the GC and BSS samples of the leptocephali

A two-step PCR employed to construct the paired-end libraries for MiSeq sequencing follows our previous study [23]. Adaptor-associated primers were used in the first PCR: 5_-ACACTCTTTCCCTACACGACGCTCTTCCGATCT + 18S30F (forward) and 5_-GTGACTGGAGTTCAGACGTGTGCTCTTCCGATCT + 18S580R (reverse). The first PCR was performed in a reaction mixture (25 μL) containing 13.5 μL H_2_O, 2.5 μL of 10 × PCR buffer, 2.5 μL dNTP (2 mM), 1.5 μL MgSO_4_ (25 mM), 1 μL template DNA, 0.5 U KOD-Plus-ver. 2 (TOYOBO, Osaka, Japan), 1.25 μL of each primer (10 μM), and 1 μL PNA (10 μM). The reaction mixture was preheated at 94°C for 3 min, followed by 30 to 40 amplification cycles (denaturation at 94°C for 15 s, annealing at 56°C for 30 s and extension at 68°C for 40 s). PCR amplification was checked using 1.5% agarose gel electrophoresis. The PCR products purified using an Agencourt MPure XP (BECKMAN COULTER, Life Sciences, Brea, California, USA) were eluted in 25 μL of TE buffer following the manufacturer protocol. The second-round PCR used the first PCR products as a template and amplified the region using primers 5’-AATGATACGGCGACCACCGAGATCTACAC- 8 bp index -ACACTCTTTCCCTACACGACGC (forward) and 5’-CAAGCA GAAGACGGCATACGAGAT- 8 bp index -GTGACTGGAGTTCAGACGTGTG (reverse). The eight base segments represent dual-index sequences used to recognize each sample; the 5’ end-sequences are adapters that allow the final product to bind or hybridize to short oligonucleotides on the surface of the Illumina flow cell; and the 3’ end-sequences are priming sites for the MiSeq sequencing. After purification, the first PCR product was diluted 10 times using Milli-Q water and used as a template for the second PCR. The second PCR was carried out in the same way as the first round of PCR, except the volume of the reaction mixture was 50 μL with the addition of 2.0 μL of the diluted PCR product. The PCR cycling conditions were as follows: initial denaturation at 94°C for 3 min, followed by 10‒12 cycles at 94°C for 15 s, 5°C for 30 s, and 68°C for 40 s. PCR amplification was again verified checked using agarose gel electrophoresis, and the PCR products were purified using an Agencourt AMPure XP (BECKMAN COULTER, USA). The amplified PCR products were quantified and the indexed second PCR products were pooled in equal concentrations and stored at ‒30°C until use for sequencing.

A PhiX DNA spike-in control was mixed with the pooled DNA library to improve the data quality of low diversity samples, such as single PCR amplicons. DNA concentrations of the pooled library and the PhiX DNA were adjusted to 4 nM using the buffer EB (10 mM Tris-HCl pH 8.5) mixed at a ratio of 7:3.5 μL. The 4 nM library was denatured with 5 μL of fresh 0.1 N NaOH. Using the HT1 buffer (provided by the Illumina MiSeq v. 2 Reagent kit for 2 × 150 bp PE), the denatured library (10 μL; 2 nM) was diluted to a final concentration of 12 pM for sequencing on the MiSeq platform.

### MPSS data treatment processes and operational taxonomic unit picking

Nucleotide sequences were demultiplexed based on the 5′-multiplex identifier (MID) tag and primer sequences using the default format in MiSeq. The sequences containing palindrome clips longer than 30 bp and homopolymer longer than 9 bp were trimmed from the sequences at both ends. The 3’ tails with an average quality score of less than 30 at the end of the last 25-bp window were also trimmed from each sequence. The 5' and 3' tails with an average quality score of <20 at the end of the last window were also trimmed from each sequence. Sequences longer than 250 bp were truncated to 250 bp by trimming the 3′ tails. The trimmed sequences shorter than 200 bp were filtered out. The demultiplexing and trimming were performed using Trimmomatic version 0.35 (http://www.usadellab.org/cms/?page=trimmomatic). The remaining sequences were merged into paired reads using Usearch version 8.0.1517 (http://www.drive5.com/usearch/). In addition, singletons were removed. Subsequently, sequences were aligned using Clustal Omega v 1.2.0. (http://www.clustal.org/omega/). Multiple sequences were aligned with each other and only sequences that were contained in more than 75% of the read positions were extracted. Filtering and a part of the multiple alignment process were performed using the screen.seqs and filter.seqs commands in Mothur, as described in the Miseq SOP (http://www.mothur.org./wiki/MiSeq_SOP) [24]. Erroneous and chimeric sequences were detected and removed using the pre.cluster (diffs = 4) and chimera.uchime (minh = 0.1; http://drive5.com/usearch/manual/uchime_algo.html) [25] commands in Mothur, respectively. Using the unique.seqs command of Mothur, the same sequences were collected into operational taxonomic units (OTUs). The contig sequences were counted as OTUs by count.seqs and used for the subsequent taxonomic identification analysis using BLASTn. Eukaryotic groups determined to be apparently of terrestrial origin were excluded, and the others were selected based on abundance.

### Statistical analysis

Sequence read counts in the sample were converted to relative read counts per million reads, which were used for principal component analysis (PCA). The number of samples having and not having a eukaryotic group was compared between the GC and BSS samples using Fisher’s exact test. Sequence read counts converted to relative read counts per million reads were subsequently standardized to logarithm. Man‒Whitney U test was used to compare the logarithms between the GC and BSS samples at α = 0.05 significance level.

## Results

### Molecular taxonomy of the leptocephali

The basic local-alignment search tool (BLAST) of the GenBank database was used to search the 16S rDNA sequences of 40 leptocephali (Table 1). The nucleotide sequences can be found in the DDBJ-EMBL-GenBank databases (LC439371‒LC439410). According to the 16S rDNA sequences, 40 leptocephali comprised 11 *A*. *japonica*, 11 *A*. *marmorata*, two *Ariosoma major*, one *Bathyuroconger* sp., one *Conger myriaster*, one *C*. *jordani* (formerly *C*. *japonicus*), one *Eurypharynx pelecanoides*, six *Gnathophis* spp., three *Gymnothorax* spp., two *Robinsia* sp., and one *Serrivomer sector*.

**Table 1:** Summary of leptocephalus samples collected in 2017 and used in this study.

ID	Sample*	BLAST top hit (% identity)^†^	date	coordinate (N, E)	BL (mm)
Aj-330	GC	*Anguilla japonica* (100)^§^	Oct. 3	18.021, 130.992	42.0
Aj-332	GC	*Anguilla japonica* (100)^§^	Oct. 3	18.021, 130.992	52.3
Aj-343	GC	*Anguilla japonica* (100)^§^	Oct. 3	18.002, 131.003	45.0
Aj-410	GC	*Anguilla japonica* (100)	Oct. 3	18.675, 131.070	42.0
Aj-446	GC	*Anguilla japonica* (100)	Oct. 4	18.024, 131.075	47.0
Aj-452	GC	*Anguilla japonica* (100)	Oct. 4	18.069, 131.068	43.0
Aj-453	GC/BSS	*Anguilla japonica* (100)	Oct. 4	18.069, 131.068	42.0
Aj-470	GC/BSS	*Anguilla japonica* (100)	Oct. 4	18.069, 131.105	44.0
Aj-475	GC/BSS	*Anguilla japonica* (99)	Oct. 4	18.059, 131.069	43.0
Aj-476	GC/BSS	*Anguilla japonica* (100)	Oct. 4	18.059, 131.069	42.0
Aj-664	GC/BSS	*Anguilla japonica* (99)	Oct. 10	18.501, 131.490	45.8
Am-38	GC	*Anguilla marmorata* (100)^§^	Sep. 30	24.501, 130.992	42.2
Am-571	GC/BSS	*Anguilla marmorata* (100)	Oct. 6	14.502, 130.988	36.7
Am-577	GC	*Anguilla marmorata* (100)^§^	Oct. 6	14.010, 131.003	37.7
Am-604	GC/BSS	*Anguilla marmorata* (100)	Oct. 8	15.490, 128.490	42.7
Am-611	GC/BSS	*Anguilla marmorata* (100)	Oct. 9	15.974, 128.502	41.1
Am-612	GC/BSS	*Anguilla marmorata* (100)	Oct. 9	15.974, 128.502	45.2
Am-697	GC	*Anguilla marmorata* (100)^§^	Oct. 11	15.601, 128.335	42.2
Am-712	GC	*Anguilla marmorata* (100)	Oct. 11	15.687, 128.369	37.6
Am-720	GC	*Anguilla marmorata* (100)	Oct. 12	15.734, 128.388	37.9
Am-736	GC	*Anguilla marmorata* (99)	Oct. 12	15.773, 128.234	51.0
Am-909	GC	*Anguilla marmorata* (100)	Oct. 14	21.810, 131.275	48.0
CG-468	BSS	*Ariosoma major* (99)^§^	Oct. 4	18.070, 131.124	65.0
CG-469	BSS	*Ariosoma major* (99)	Oct. 4	18.070, 131.124	214.0
CG-305	GC	*Bathyuroconger vicinus* (96)^§^	Oct. 2	18.506, 131.006	57.7
Cm-342	GC	*Conger myriaster* (99)^§^	Oct. 3	18.021, 130.992	65.0
Cj-488	GC/BSS	*Conger jordani* (100)^§^	Oct. 4	18.059, 131.069	43.1
EU-758	GC	*Eurypharynx pelecanoides* (99)^§^	Oct. 12	15.313, 128.234	18.1
CG-15	GC	*Gnathophis bathytopos* (99)^§^	Sep. 29	25.004, 130.985	62.7
CG-16	GC	*Gnathophis bathytopos* (98)^§^	Sep. 29	25.004, 130.985	49.6
CG-301	GC	*Gnathophis bathytopos* (99)^§^	Oct. 2	18.506, 131.005	56.3
CG-303	GC	*Gnathophis bathytopos* (99)^§^	Oct. 2	18.506, 131.005	62.8
CG-878	GC	*Gnathophis bathytopos* (99)^§^	Oct. 13	21.424, 131.143	67.5
CG-879	GC	*Gnathophis bathytopos* (99)^§^	Oct. 13	21.424, 131.143	68.1
MR-344	GC	*Gymnothorax melatremus* (92)	Oct. 3	18.002, 131.003	42.0
MR-471	BSS	*Gymnothorax margaritophorus* (100)^§^	Oct. 4	18.069, 131.101	34.0
MR-483	GC/BSS	*Gymnothorax niphostigmus* (93)^§^	Oct. 4	18.059, 131.069	41.6
CH-572	GC/BSS	*Robinsia catherinae* (96)^§^	Oct. 6	14.502, 130.988	69.3
CH-663	GC/BSS	*Robinsia catherinae* (96)^§^	Oct. 9	18.485, 131.001	63.8
SR-614	BSS	*Serrivomer sector* (99)^§^	Oct. 9	15.974, 128.502	45.5

*GC: only gut content sample was analyzed; GC/BSS: both gut content and body surface scraping samples were analyzed; BSS: only body surface scraping sample was analyzed.

^†^based on mitochondrial 16S rDNA sequence analysis for the leptocephali.

^§^leptocephali were subjected to direct 18 rDNA sequence analysis.

### Overview of eukaryotic groups in the GC and BSS samples

The GC sample was obtained from 36 leptocephali, because squeezing of GC failed in four samples (Table 1). The BSS sample was collected from 17 leptocephali (Table 1). Of OTU obtained after quality check for 18S rDNA sequences, those having low similarity (< 90%) with the top BLASTn hit sequences (10 OTUs comprising 3484 reads) were removed, resulting in 29 eukaryotic groups and 154 OTUs comprising 269185 reads (106 OTUs comprising 162897 reads in the GC sample and 101 OTUs comprising 106288 reads in the BSS sample) (Table 2). Five eukaryotic groups (asterisk in Table 2) determined to be contaminants of terrestrial origin in the laboratory and fifteen eukaryotic groups (double dagger in Table 2) occurring at low read frequency (<1%) were excluded from further analysis. Phylogenetic analysis using seven fish OTUs with their top BLASTn hits and the host 18S rDNA sequences determined using direct nucleotide sequencing (accession No. LC464077‒LC464098) indicated that almost all fish 18S rDNAs obtained using NGS were of the host, revealing incomplete PNA clamping. Therefore, fish OTUs comprising 27507 reads (7 OTUs comprising 26880 reads in the GC sample and 4 OTUs comprising 627 reads in the BSS sample) were also excluded (pilcrow in Table 2). Remaining after applying these selection criteria were eight eukaryotic groups comprising jellyfish (Cnidaria) (21 OTUs, 99258 reads), conoid parasite (Conoidasida) (7 OTUs, 26272 reads), tunicate (Chordata) (14 OTUs, 24148 reads), copepod (Copepoda) (15 OTUs, 14144 reads), krill (Euphausiacea) (11 OTUs, 11800 reads), annelid (Polychaeta) (6 OTUs, 1469 reads), fungus (18 OTUs, 20452 reads), and dinoflagellate (Dinophyceae) (6 OTUs, 4017 reads) (Table 2). The nucleotide sequences of these 98 OTUs and seven fish OTUs generated using NGS are available in the DDBJ-EMBL-GenBank database (LC474264‒LC474368). Raw read count data for the eight eukaryotic groups are available in S1 Table. One GS sample and one BSS sample having none of these eukaryotic groups were excluded from further analyses.

**Table 2:** Summary of eukaryotic groups detected in the gut content (GC) and body surface scraping (BSS) samples of eel leptocephali, and number of OTUs and reads of 18S rDNA.

				No. OTUs			No. reads (No. larvae)		BLAST
	organism group	Phylum	lower taxa	all	GC	BSS	GC	BSS	% identity
metazoa	jellyfish	Cnidaria	Anthozoa, Hydrozoa	21	18	16	34171 (18)	65087^†^ (13^†^)	96.6–100
	conoid parasite	Apicomplexa	Coccidia, Gregarinasina	7	7	0	24672^†^ (17^†^)	0 (0)	90.9–98.5
	tunicate	Chordata	Appendicularia, Thaliacea	14	12	14	10451 (13)	13697 (6)	95.9–100
	copepod	Arthropoda	Copepoda	15	11	9	11460 (16)	2684 (7)	92.3–100
	krill	Arthropoda	Euphausiacea	11	9	10	4068 (17)	7732 (13^†^)	96.9–99.6
	annelid	Annelida	Polychaeta	6	1	6	19 (1)	1450^†^ (5^†^)	97.8–100
	fish^¶^	Chordata	Actinopterygii	7	7	4	26880 (33)	627 (12)	91.8–99.8
	acorn worm^‡^	Hemichordata	Enteropneusta	2	2	2	412 (2)	475 (2)	98.0–99.8
	shrimp^‡^	Arthropoda	Decapoda	4	1	3	724 (1)	130 (2)	99.6–100
	snail^‡^	Mollusca	Gastropoda	9	2	7	197 (3)	403 (5)	94.0–99.8
	arrow worm^‡^	Chaetognatha	Aphragmophora	4	4	3	50 (7)	54 (6)	98.7–99.6
	comb jelly^‡^	Ctenophora	Tentaculata	2	0	2	0 (0)	101 (3)	100
	ostracods^‡^	Arthropoda	Halocyprida	1	1	1	22 (3)	24 (1)	99.5
	mite*	Arthropoda	Arachnida	1	1	1	22748 (13)	5822 (4)	99.8
	public lice*	Arthropoda	Insecta	1	1	1	2069 (3)	1 (1)	100
	silkworm*	Arthropoda	Insecta	1	1	0	1064 (1)	0 (0)	99.6
	human*	Chordata	Mammalia	1	1	0	8 (2)	0 (0)	99.5
non-metazoa	fungi	Fungi		18	16	4	17156^†^ (17^†^)	3296 (2)	95.1–100
	dinoflagellate	Dinoflagellata	Gonyaulacales, Syndiniales	6	1	5	1467 (1)	2550 (5^†^)	91.1–99.8
	radiolaria^‡^	Radiozoa	Collodaria	6	3	3	919 (2)	88 (3)	91.8–99.8
	golden algae^‡^	Chrysophyceae	Chromulinales	1	1	0	816 (2)	0 (0)	100
	green algae^‡^	Chlorophyta	Prasinococcales, Pyramimonadales	2	0	2	0 (0)	497 (3)	91.1–99.6
	filose amoebae^‡^	Cercozoa	Chlorarachniophyceae	1	0	1	0 (0)	138 (1)	99.6
	heterokonts^‡^	Bigyra	Bicoecida	2	0	2	0 (0)	97 (2)	100
	cryptomonads^‡^	Cryptophyta	Pyrenomonadales	1	0	1	0 (0)	93 (1)	91.7
	heterokont algae^‡^	Dictyochophyceae	Rhizochromulinales	1	0	1	0 (0)	68 (1)	97.5
	apusozoa^‡^	Apusozoa	Apusomonadida	1	0	1	0 (0)	17 (1)	95.4
	brown algae^‡^	Phaeophyceae	Laminariales	1	0	1	0 (0)	2 (1)	97.7
	flowering plants*	Magnoliophyta		7	6	1	3522 (3)	37 (1)	94.6–100
		total No. OTUs and reads of all taxa		154	106	101	162897	106288	
		No. OTUs and reads of selected taxa		98	75	64	103464	97612	

^¶^determined to be the host 18S rDNA

^‡^excluded due to the low read frequency (<1%)

*determined to be terrestrial origin and excluded

^†^significantly greater than the other sample.

**Fig 1:**
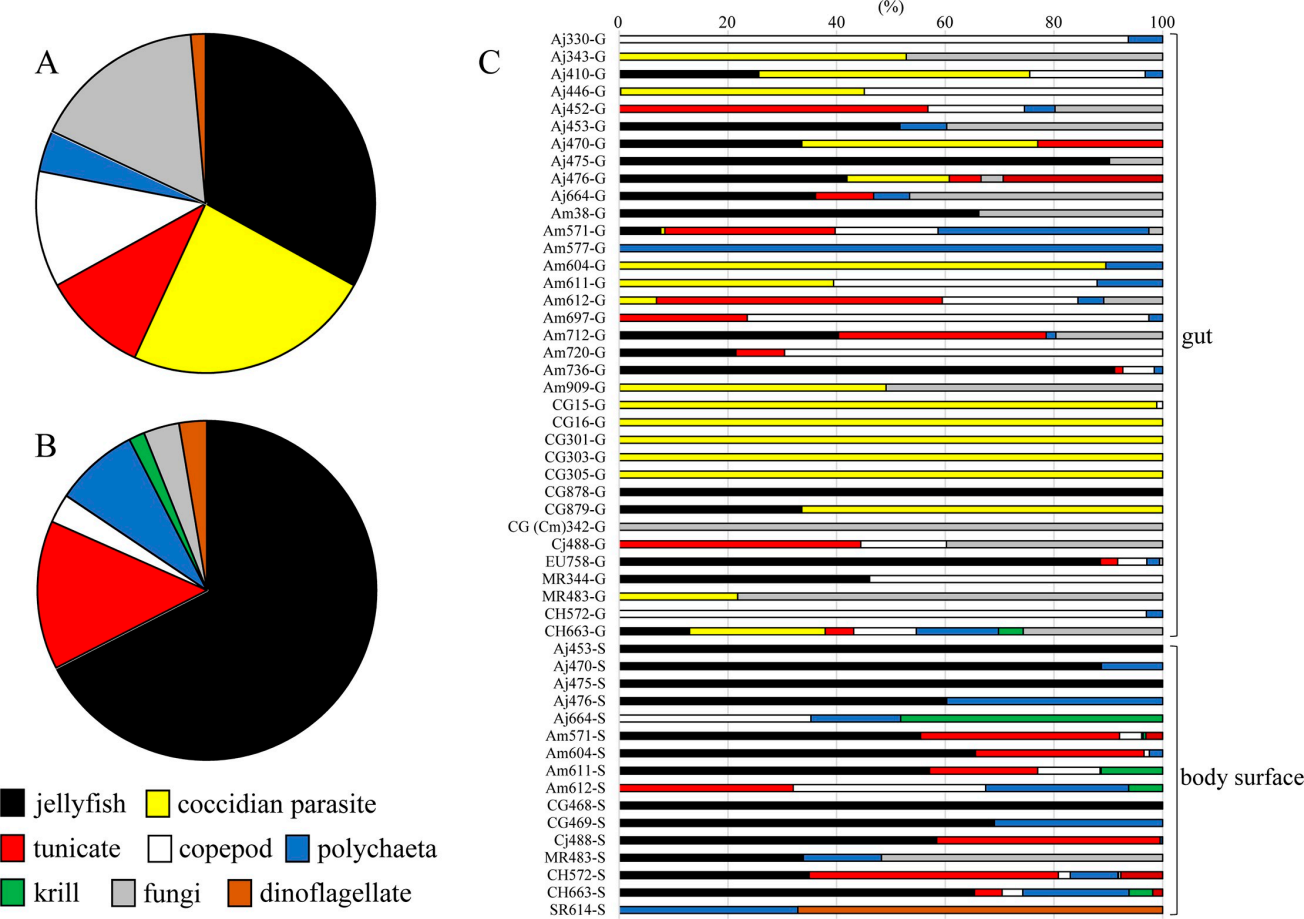
Composition of the eight eukaryotic groups (see Table 2) detected in the gut and body surface samples of Anguilliformes leptocephali. (A) Overview of the eight eukaryotic groups in the gut samples (n = 35). (B) Overview of the seven eukaryotic groups in the body surface samples (n = 16). (C) Composition of the eight eukaryotic groups in each leptocephalus.

Eight eukaryotic groups detected in the GC samples comprised 75 OTUs and 103464 reads, and seven eukaryotic groups detected in the BSS samples comprised 64 OTUs and 97612 reads (Table 2, Fig 1A and 1B). Jellyfish was the primary component in both samples, occupying 33.0% of total reads in the GC samples and 67.5% in the BSS samples. Conoid parasite was the second-most abundant contributor (23.8%) in the GC sample but zero in the BSS sample. Less frequent eukaryotic groups in the GC and BSS samples were tunicate (10.1% and 14.0%, respectively), copepod (11.1% and 2.7%), krill (3.9% and 7.9%), annelid (< 0.1% and 1.5%), fungus (16.6% and 3.4%), and dinoflagellate (1.4% and 2.6%).

### Eukaryotic compositions in each sample

**Fig 2:**
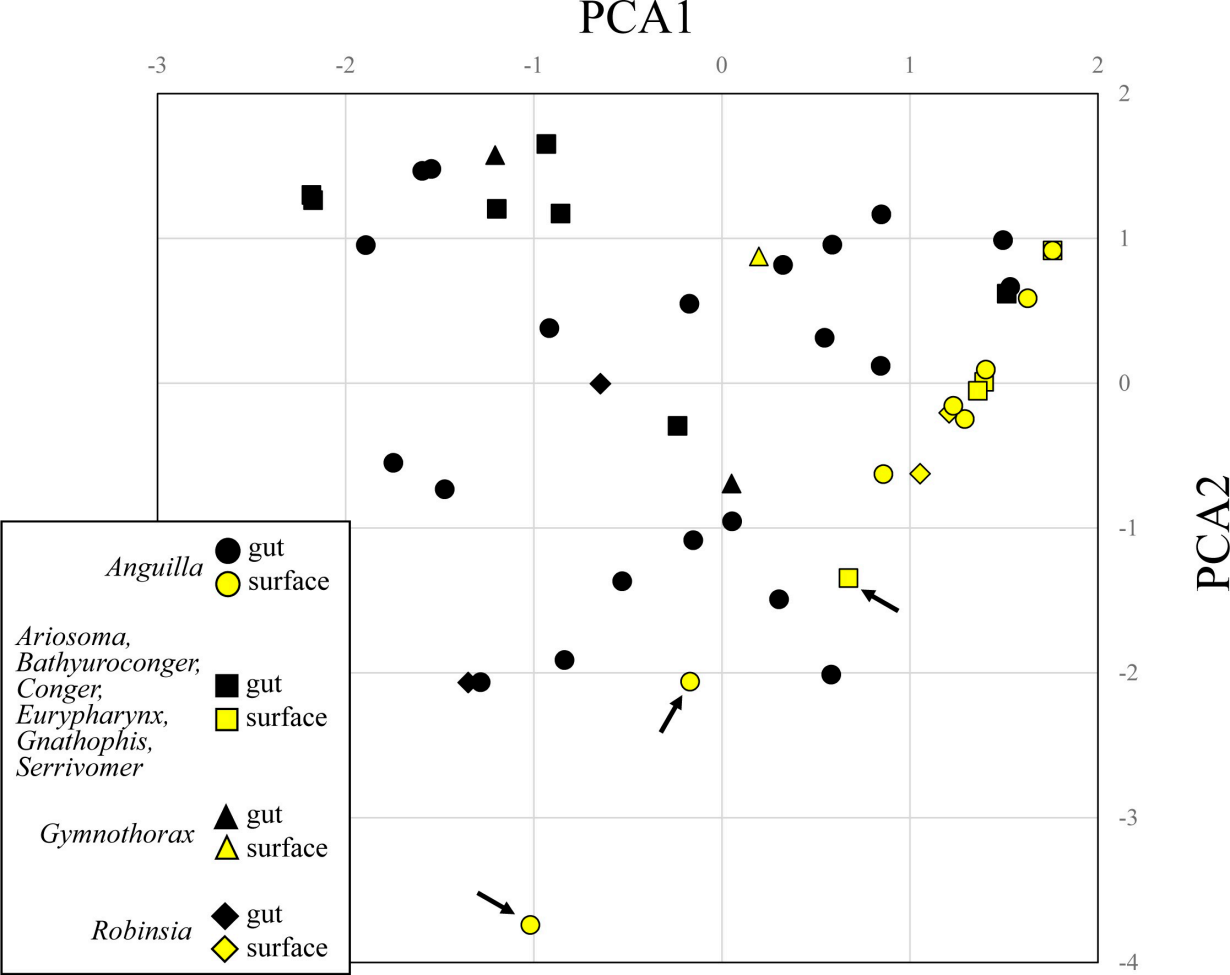
Principal component analysis of the eukaryotic 18S rDNA compositions in the gut contents (black symbols) and body surface scraping samples (yellow symbols). Circles (*Anguilla japonica* and *A*. *marmorata*), triangles (*Gymnothorax* spp.), diamonds (*Robinsia* sp.), and squares (other species). Arrows indicate three outliers in the body surface scraping samples having no cnidarian read.

Eukaryotic composition considerably varied among leptocephalus individuals (Fig 1C). A Shannon-Wiener diversity index ranged between 0 and 0.814 in the GC samples and between 0 and 0.699 in the BSS samples with no significant difference between the samples (Mann‒Whitney U test, *p* = 0.578), but heterogeneity between the GC and BSS samples mentioned above was also the case at individual level (Fig 1C). Systematic difference in eukaryotic compositions between the GC and BSS samples was illustrated using PCA analysis (Fig 2). The GC samples were dispersed regardless of species (Fig 2, black symbols). On the other hand, the BSS samples were closely related one another (Fig 2, yellow symbols) except for three outliers (Fig 2, arrow), in which no jellyfish read was observed in these three BSS samples (see also Fig 1C, Aj-664S, Am-612S, and SR-614S).

Jellyfish reads were detected in 18 of 35 GC samples and 13 of 16 BSS samples with significant difference (Fisher’s exact test, *p* = 0.040), and the standardized read counts of the jellyfish were greater in the BSS samples than in the GC samples (Mann–Whitney U test, *p* < 0.005). Occurrences of the krill, annelid, and dinoflagellate were also greater in the BSS samples than in the GC samples (Fisher’s exact test, *p* < 0.05), of which the standardized read counts of the annelid were greater in the BSS samples than in the GC samples (Mann–Whitney U test, *p* = 0.026). In contrast, occurrences of the conoid parasite and fungus were significantly higher in the GC samples than in the BSS samples; specifically no conoid parasite read was detected in the BSS samples (Fisher’s exact test, *p* < 0.05, Mann–Whitney U test, *p* < 0.05). No such significant heterogeneity between the GC and BSS samples was observed in the tunicate and copepod.

**Fig 3:**
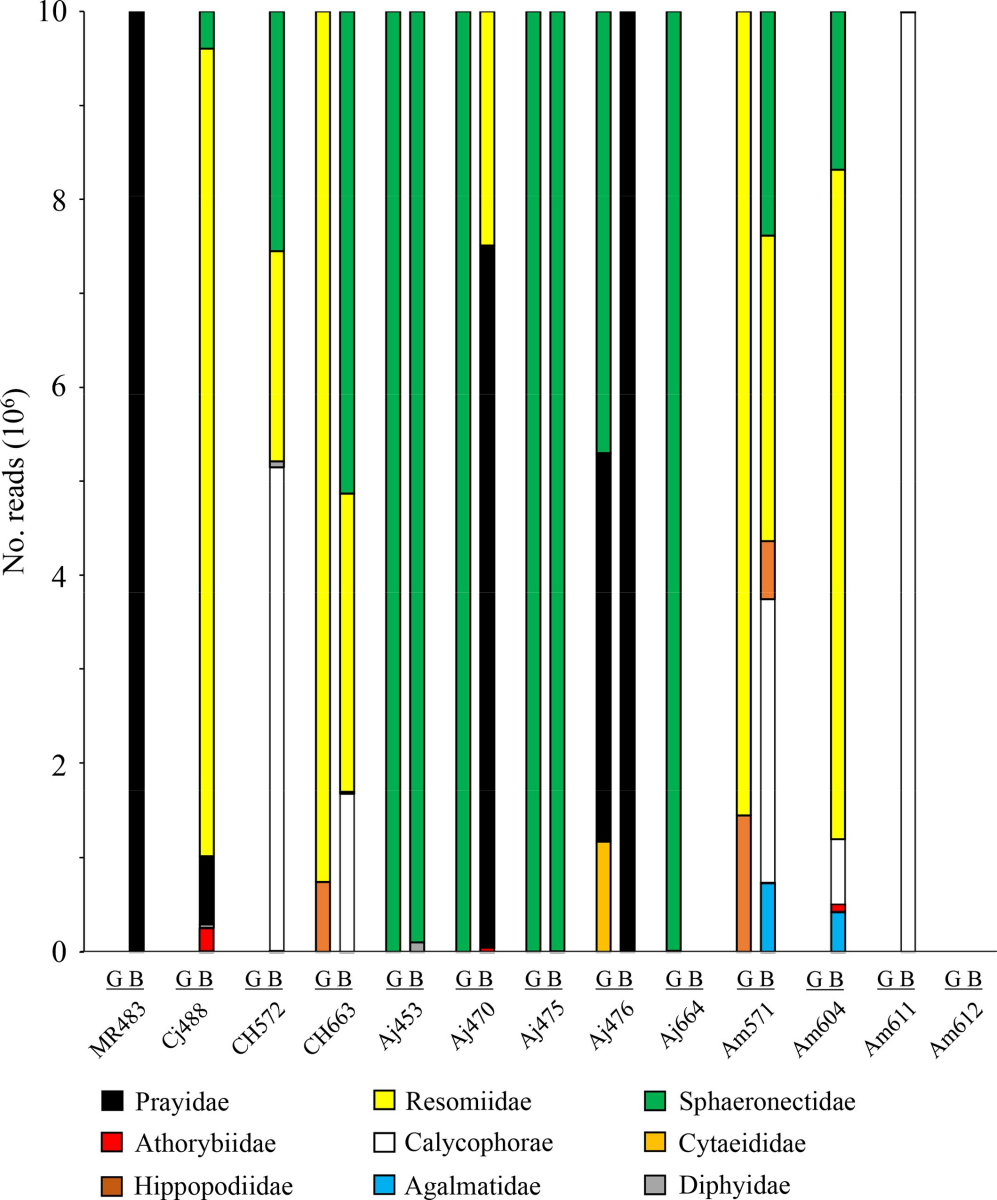
Composition of cnidarian jellyfish taxa detected in the gut content (G) and body surface scraping (B) samples of 13 leptocephali having both the G and B samples. Sequence read counts in the sample were converted to relative read counts per million reads.

Since it has been suggested that cnidarian jellyfishes may be important diet for the leptocephalus larvae [12, 14], jellyfish taxa and the read number in 13 leptocephali having both the GC and BSS samples were investigated (Fig 3). Eight families and one suborder (Calycophorae) of cnidarian taxa were chosen according to the abundancy (total read number larger than 100). Of 13 leptocephali, six had jellyfish reads in both the GC and BSS samples, five had those only in the BSS sample, one had those only in the GC sample, and one had no jellyfish read in both the GC and BSS samples. Of six leptocephali having jellyfish reads in both the GC and BSS samples, only one (Aj470) had no common jellyfish read between the GC and BSS samples.

## Discussion

Since primer choice has been known to considerably influence quantitative estimations on the target molecules in metagenome study [26], results of our metagenomic analysis may not reflect the true eukaryote composition in the gut contents of eel leptocephali. However, our results are reliable in comparing the eukaryote composition between the GC and the BSS samples of the eel leptocephali. We observed that almost all eukaryotic groups (except for conoid parasite) highlighted were common between the GC and BSS samples. The occurrence of jellyfish, krill, annelid, and dinoflagellate and/or the standardized read counts were significantly higher in the BSS samples than in the GC samples. Abundant eukaryotic groups, such as conoid parasite and fungi, were distinctively observed in the GC samples than in the BSS samples, indicating little index hopping effect in this study. Compared with the BSS samples, conoid parasite exclusively detected in the GC samples may be indicative of very little cross contamination in the direction from the GC samples to the BSS samples. One dinoflagellate OTU assigned to *Hematodinium perezi* was observed only in the GC samples; this dinoflagellate is also known to be parasitic [27]. Therefore, the results obtained in this study indicate that just careful rinsing of the body surface of the leptocephali with sterile artificial seawater cannot prevent cross contamination of DNAs derived from the body surface and/or environment.

Cnidarian planktons were suggested to be an important diet for the leptocephalus larvae, because 18S rDNA sequences of hydrozoa jellyfish predominated the GC of *A*. *anguilla* larvae [12,14]. However, there are several discrepancies in judging that jellyfish is the main food source for leptocephali. Data accumulating from stable isotope analysis indicate that cnidarians usually stay at relatively higher trophic position [28–36]; trophic positions of leptocephali have been observed to be apparently lower than those of cnidarian jellyfish [6, 18]. A few cnidarians may stay at low trophic positions [28,35], and it was claimed that cnidarians in oligotrophic areas, like the Sargasso Sea, may have low δ^15^N values [14]. However, it is unlikely that leptocephali selectively consume specific cnidarians at lower trophic position and migrate in oligotrophic water mass with oligotrophic organisms all the way down from the spawning area to nursery area. Because genes from gelatinous zooplankton have been found in the guts of lobster larvae [22,37–39], jellyfish consumption by leptocephali was further advocated owing to similarity in the highly flattened body and long larval period between the lobster and eel larvae [14]. The lobster larvae are active predator, because they have actually been observed to capture and prey upon a variety of agile zooplanktons [37,40,41]; however, such predatory behavior toward zooplankton has never been observed in leptocephali [5,42,43]. Results of the feeding experiments of leptocephali attempted till date are summarized in Table 3. Hatchery-produced *A*. *japonica* or *A*. *anguilla* leptocephali were used in all experiments except for wild-caught leptocephali of pike conger (*Muraenesox cinereus*) and white-spotted conger (*Conger myriaster*) [5]. Among the considerably wide variety of food candidates tested, all well consumed foods, except for the smallest aloricate rotifer *Proales similis*, were processed in the form of a paste. Gelatinous animals appear as an unfavorable feed, because fresh chopped Cnidaria (*Aurelia* sp.) was still capable of killing the Japanese eel leptocephali by the nematocysts and alive ctenophore even captured and preyed upon the leptocephali (unpublished laboratory experiment by Hideki Tanaka). Furthermore, Japanese eel leptocephali teeth were disadvantage for feeding on sticky jellyfish tissue (unpublished on board experiment by Seinen Chow). Therefore, we conclude that cross contamination from the body surface occurred while collecting the GC and the pronounced detection of cnidarian 18S rDNA from the body surface was due to the cnidarian nematocysts sting to the leptocephali in the plankton net. No notable difference was found in eukaryotic composition between full and empty gut individuals [14], which also supports our conclusion.

**Table 3:** Summary of feeding experiments of the leptocephalus larvae.

	zooplankton	phytoplankton	animal tissue	bacteria	artificial diets
consumed well	*Proales similis* [42]		paste of squid [5], paste of *Proales similis* [50]		paste using shark egg [51, 52], Indipe-plus^φ^ (paste using Labyrinthulea and krill powder)^§^
consumed a little	rotifer [42, 48], Larvacea and larvacean house [53], eggs of sea urchin, *Anguilla* and sea bream^†^, *Proales similis* [50]	dinoflagellate^†^, microalgal cells [53], small-size plankton^†^^,^ [49], Labyrinthulea cells^§^	powder of freeze-dried squid, mussel gonad and shrimp^†^, chicken egg yolk^†^, paste of rotifer [43], feces of *Ruditapes philippinarum* and *Artemia*^§^	bacterial flocks in oyster and krill extracts^§^	ITOMEITO* (glass eel feed) ^†^, TEP produced by microalgae [53], *Ascophyllum nodosum* extract^§^, macro algal detritus produced by enzyme treatment^§^
not consumed	copepod larvae [5, 42], ctenophora [5], *Artemia* nauplii, Larvacea and larvacean house^†^, Sargasso plankton [43]	*Nannochloropsis* and DHA-enriched *Euglena*^†^	powder of freeze-dried salted jellyfish, ray fin and eel leptocephalus^†^, paste of fish, jellyfish, ctenophora, polychaeta, shrimp and snail^‡^, feces of *Turbo sazae*^§^	photosynthetic bacteria^†^	algal extract digested by bacteria^†^, mince of gelatin^†^, Weider in jelly^¶^^,^^§^, CalorieMate fluid^⁋^^,^^§^, ornamental fish feed^§^
harmful	eggs of sea star and sea cucumber^†^, ctenophora^†^^,^^‡^	large-size planktons [49], *Euglena gracilis* cultured with glucose^§^	chopped cnidaria^†^^,^^‡^		

^†^unpublished laboratory experiment (by Hideki Tanaka)

^‡^unpublished on board experiment (by Seinen Chow)

^§^unpublished laboratory experiment (by Tsutomu Tomoda)

^φ^Scientec Co., Ltd.

*Marubeni Nisshin Feed Co., Ltd.

^¶^Morinaga & Co., Ltd.

^⁋^Otsuka Pharmaceutical Co.

Regarding the eukaryotes that were found in the gut of leptocephali, parasites are definitive and fungi are probably positive candidates; however, it is unlikely that these can be feed for the leptocephali. Not many, but feces of zooplankton, larvacean and their houses have been occasionally observed in the gut of leptocephali [6–10], corresponding to relatively larger read counts of copepod and tunicate 18S rDNA in the gut samples observed in this study. Thus, the present study supports previous results that leptocephali utilize detritus materials, so called marine snow [6,8–10,13,17,19,44]. In the tropical and subtropical regions of the western North Pacific, copepods are reported to be the most abundant zooplankton taxa occupying at least 70% or more of total mesozooplankton biomass in mesopelagic layer, followed by urochordates and chaetognaths but cnidarians jellyfish at much lower abundance [45–47]. Cnidarians might contribute to some part of the marine snow formation and therefore, may be a diet component for the leptocephali. However, it is unlikely that cnidarians are the main food sources for the eel leptocephali because of the higher stable isotope ratio and lower abundance.

## Supporting information

S1 Table Eukaryotic groups highlighted in this study. Raw read counts of eight eukaryotic groups detected in 36 gut contents (GC) and 17 body surface scraping (BSS) samples obtained from 40 Anguilliformes leptocephali.
(XLSX)
